# A reverse Monte Carlo algorithm to simulate two-dimensional small-angle scattering intensities^1^

**DOI:** 10.1107/S1600576722009219

**Published:** 2022-11-29

**Authors:** Lester C. Barnsley, Nileena Nandakumaran, Artem Feoktystov, Martin Dulle, Lisa Fruhner, Mikhail Feygenson

**Affiliations:** a Australian Synchrotron, ANSTO, Clayton 3168, Australia; b Forschungszentrum Jülich GmbH, Jülich Centre for Neutron Science (JCNS) at Heinz Maier-Leibnitz Zentrum (MLZ), 85748 Garching, Germany; c Forschungszentrum Jülich GmbH, Jülich Centre for Neutron Science (JCNS-2) and Peter Grünberg Institut (PGI), JARA-FIT, 52425 Jülich, Germany; d Lehrstuhl für Experimentalphysik IVc, RWTH Aachen University, 52056 Aachen, Germany; e Forschungszentrum Jülich GmbH, Jülich Centre for Neutron Science (JCNS-1), 52425 Jülich, Germany; f European Spallation Source ERIC, SE-22100 Lund, Sweden; Universität zu Köln, Germany

**Keywords:** small-angle neutron scattering, small-angle X-ray scattering, magnetic nanoparticles, superparamagnetic iron oxide nanoparticles, reverse Monte Carlo simulations

## Abstract

Small-angle scattering (SAS) experiments are a powerful method for studying self-assembly phenomena in nanoscopic materials because of the sensitivity of the technique to structures formed by interactions on the nanoscale. Numerous out-of-the-box options exist for analysing structures measured by SAS but many of these are underpinned by assumptions about the underlying interactions that are not always relevant for a given system. Here, a numerical algorithm based on reverse Monte Carlo simulations is described to model the intensity observed on a SAS detector as a function of the scattering vector. The model simulates a two-dimensional detector image, accounting for magnetic scattering, instrument resolution, particle polydispersity and particle collisions, while making no further assumptions about the underlying particle interactions. By simulating a two-dimensional image that can be potentially anisotropic, the algorithm is particularly useful for studying systems driven by anisotropic interactions. The final output of the algorithm is a relative particle distribution, allowing visualization of particle structures that form over long-range length scales (*i.e.* several hundred nanometres), along with an orientational distribution of magnetic moments. The effectiveness of the algorithm is shown by modelling a SAS experimental data set studying finite-length chains consisting of magnetic nanoparticles, which assembled in the presence of a strong magnetic field due to dipole interactions.

## Abstract 2

Small-angle neutron scattering and small-angle X-ray scattering are important experimental techniques for studying the behaviour and properties of materials on the nanoscale. This article describes a numerical algorithm that uses reverse Monte Carlo simulations to model scattering intensities observed on a two-dimensional small-angle scattering detector.

## 1. Introduction

Small-angle neutron scattering (SANS) and small-angle X-ray scattering (SAXS) are important experimental techniques for studying the behaviour and properties of materials on the nanoscale. Small-angle scattering (SAS) has been used to investigate systems relevant to a range of scientific fields, including proteins (Bizien *et al.*, 2016 ▸; Mahieu & Gabel, 2018 ▸; Brosey & Tainer, 2019 ▸), polymers (Mortensen, 2001 ▸; Jaksch *et al.*, 2016 ▸; Papadakis *et al.*, 2019 ▸; Wei & Hore, 2021 ▸), micelles (Das *et al.*, 2012 ▸; Kelly *et al.*, 2019 ▸), inorganic nanoparticles (Mehdizadeh Taheri *et al.*, 2015 ▸; Fu *et al.*, 2016 ▸; Bender *et al.*, 2017 ▸; Krycka *et al.*, 2018 ▸), battery materials (He *et al.*, 2017 ▸) and magnetic vortices (Mühlbauer *et al.*, 2009 ▸; Demirdiş *et al.*, 2016 ▸). While the technique is well established for its versatility and compatibility with a range of sample environments for *in situ* studies, analysis of experimentally acquired data is still challenging, particularly in light of the growing complexity of the structures and their temporal and spatial arrangements, which can be readily affected by external parameters.

The conventional approach to analysis of SAS results is to fit experimental data with a set of relevant semi-analytical models to infer the structural properties and extract physical parameters about the measured ensemble. A huge range of out-of-the-box options for fitting models to SAS data is readily available and this approach is highly effective for simple systems, particularly when the measured sample consists of a highly monodisperse suspension of particles with minimal aggregations. However, this approach can only provide limited information from complex systems, particularly systems involving particles that consist of multiple phases and/or geometries, or assemblies that are partially ordered or anisotropic. The variety of form factors and structure factors typically available in modelling software often require assumptions that may not be relevant to a given system (*e.g.* isotropic distributions, well described interaction potentials or long-range order).

Numerical techniques are becoming increasingly popular (and necessary) to fully describe the non-trivial particle geometries and structures that are investigated by SAS (Frenkel *et al.*, 1986 ▸; Svergun & Koch, 2003 ▸; Olds & Duxbury, 2014 ▸; Honecker *et al.*, 2020 ▸). Numerical analysis of SAS data can be helpful to defer or even bypass many of the assumptions required by semi-analytical models. Numerical techniques for SAS analysis can be broadly divided into two categories (Olds & Duxbury, 2014 ▸). The first uses reverse Monte Carlo simulations, in which particle positions and properties are freely distributed inside a physical space with fixed boundaries and treated as separate scattering sites (Pedersen *et al.*, 2003 ▸; Musino *et al.*, 2018 ▸). The second approach is based on either discrete or fast Fourier transforms, in which a physical distribution is cast to a real-space grid and then transformed into a scattering intensity profile in reciprocal space (Schmidt-Rohr, 2007 ▸; Bender *et al.*, 2017 ▸). The relative advantages of both approaches have been debated but, in simplistic terms, reverse Monte Carlo simulations are more effective for modelling structure factors from assemblies of simple particles, while Fourier transforms are proficient for solving form factors of advanced non-trivial particle geometries.

Conventionally, analysis of SAS data has been focused on the radially averaged intensity profiles from isotropic systems, but experimental detectors are typically two dimensional, making them sensitive to two components of the scattering vector, and, therefore, anisotropic intensity distributions that are projected in the detector plane. Fully accounting for the two-dimensional detector image is particularly important in experiments studying magnetic systems, as the externally applied field defines an axis of anisotropy. In particular, SANS is sensitive to magnetic scattering, which is enhanced by leveraging neutron polarization, either by polarizing the incident neutron beam (polarization) and/or by filtering by the neutron spin, post-scattering (analysis). The scattering cross sections of the various polarization analysis SANS spin channels are well known in certain cases, *e.g.* weakly inhomogeneous ferromagnets or well dispersed two-phase magnetic systems [see Michels (2014 ▸) for a detailed discussion], and the data analysis often focuses on describing the angle dependence of the observed intensity (Oberdick *et al.*, 2018 ▸; Ijiri *et al.*, 2019 ▸; Zákutná *et al.*, 2020 ▸). However, in the presence of interacting particles with non-trivial structure factors, the conventional analysis is not straightforward.

In this article, we introduce an algorithm that uses reverse Monte Carlo simulations to model scattering intensities observed by a two-dimensional detector. The algorithm considers magnetic scattering, finite instrument resolution and the individual properties of particles (including polydispersity and magnetization direction), making no assumptions about the underlying particle interactions except to exclude collisions between particles. While the algorithm performs best for modelling ensembles of particles with well described form factors (*i.e.* collections of spherical nanoparticles), functions exist to simulate scattering contributions for more advanced particles with non-trivial geometries by utilizing discrete Fourier transforms.

The main application is to model scattering profiles with structure factors that are either anisotropic or partially disordered. This approach was used to successfully model results from a set of SANS experiments studying nanochain formation from iron oxide nanoparticles induced by magnetic interactions (Nandakumaran *et al.*, 2021 ▸). The output of the model is a set of real-space particle configurations, including magnetic moment orientations, which can be visualized and further analysed to make inferences about physical distributions and magnetic ordering that are consistent with the observed scattering intensity. The capability for the algorithm to model polarized SANS (SANSPol) data is also demonstrated. The algorithm is written using Python 3, a modern open-source object-orientated programming language, in order to maximize flexibility and extendibility, and is underpinned by open-source packages that are readily available on the Python Package Index. The physics simulated by the algorithm is an extension of the concept described by Musino *et al.* (2018 ▸), in which reverse Monte Carlo simulations are used to model the structure factor observed in one-dimensional scattering profiles from aggregations of surface-coated silica nanoparticles.

## 2. Methods

### 2.1. Scattering simulation

The scattering cross section of an ensemble of *N* non-magnetic particles in a box with volume *V* is given by 

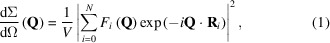

where **Q** = (*Q*_*x*_, *Q*_*y*_, *Q*_*z*_) is the scattering vector, *F*_*i*_ is the form amplitude of an individual particle *i* associated with its form factor and **R**_*i*_ = (*x*_*i*_, *y*_*i*_, *z*_*i*_) is the position of the particle. For advanced particle geometries, the form amplitude can be numerically calculated and broadcast (or ‘mapped’) to **Q**, but in the case of simple particles (*e.g.* radially symmetric particles), an analytical expression for the function *F*_*i*_(**Q**) can be provided. For example, in the general case of a radially symmetric particle, 

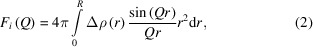

where *R* is the radius of the particle, Δρ(*r*) is the contrast between the particle and solvent scattering length densities (SLD) at distance *r* from the centre of the particle, and *Q* is the magnitude of the scattering vector. In the specific case of a core–shell particle, this is 

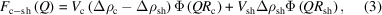

where *R*_c_ is the radius of the core, 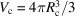 is the volume of the core, *V*_sh_ = 4π(*R*_c_ + *t*_sh_)^3^/3 is the volume of the overall particle, *t*_sh_ is the thickness of the shell, Δρ_c_ is the contrast between the core and solvent SLD values, Δρ_sh_ is the contrast between the shell and solvent SLD values, and 

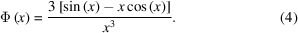

More advanced particle geometries may be considered numerically. To save computation time, we leverage the fact that, conventionally, a SANS detector is sensitive to two dimensions of the scattering vector, *Q*_*x*_ and *Q*_*y*_, with *Q*_*z*_ set to 0. This allows separation of the *z* dependency of Δρ(*x*, *y*, *z*), so that 

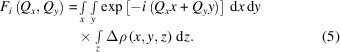

The two separate integrals imply two separate numerical summations. The integral on the second line involves summing the SLD along the *z* axis to the *xy* plane, which is performed first. The second step is then to broadcast the resultant sum to *Q*_*x*_ and *Q*_*y*_, which can be computed as either a discrete or a fast Fourier transform. Much of the computation advantage is obtained by caching the form amplitude and the result of 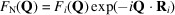 as arrays in *NumPy* (an open-source Python library for working with numerical arrays; https://numpy.org/; Harris *et al.*, 2020 ▸), where *F*_N_ is referred to as the nuclear scattering amplitude.

Magnetic scattering is calculated in an analogous way, except that **F**_M_(**Q**) = *b*_H_ *V*_M_ **M**(**Q**) is a vector with three spatial components. Here **M**(**Q**) = [*M*_*x*_(**Q**), *M*_*y*_(**Q**), *M*_*z*_(**Q**)] is the Fourier transform of the magnetization vector **M**(**r**) = [*M*_*x*_(**r**), *M*_*y*_(**r**), *M*_*z*_(**r**)], *b*_H_ = 2.91 × 10^8^ A^−1^ m^−1^ is a conversion constant for the magnetic scattering length (derived from other physical constants) (Honecker & Michels, 2013 ▸; Mühlbauer *et al.*, 2019 ▸) and *V*_M_ is the scattering volume of a magnetic particle. Due to the nature of the neutron interaction with the internal field, only the magnetization component perpendicular to the scattering vector is detected, resulting in 

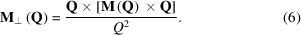

A given component (in this case, the *y* component) of the magnetic form amplitude for a spherical particle, *i*, in a non-magnetic solvent is calculated by 

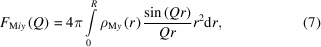

where ρ_M*y*_(*r*) = *b*_H_ *M*_*y*_(*r*) is the magnetic SLD. The particle position is encoded by multiplying by 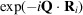 , analogously to equation (1), and the other components of **F**_M*i*_(**Q**) are computed by following the same method, to obtain the individual particle contributions. The individual particle contributions are summed to obtain the total amplitudes, which are processed analogously to equation (6) to obtain the components of the magnetic amplitude perpendicular to the scattering vector: *F*_⊥M*x*_, *F*_⊥M*y*_ and *F*_⊥M*z*_.

In an experiment using polarized neutrons, the contribution of the magnetic scattering to the final detector signal can be controlled by spin filtering, either before sample scattering by using an incident-beam polarizer and/or after sample scattering by using neutron-spin filters. For simplicity, we consider the case where the sample field is aligned with the *y* axis and the beam direction is aligned with the *z* axis, and we leave correction of spin inefficiencies as a step for data reduction. The four polarization analysis cross sections [based on the Moon, Riste and Koehler equations (Moon *et al.*, 1969 ▸)] are 

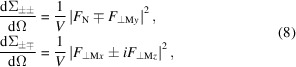

for the non-spin-flip and spin-flip signals, respectively. The ‘spin down’ and ‘spin up’ SANSPol intensities are obtained by summing the above cross sections with the same incident polarization state so that 

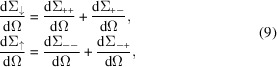

and the unpolarized cross section is recovered by adding the SANSPol intensities: 

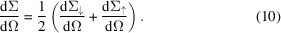

Here, ‘spin up’ is used to indicate a spin flipper in the on state, equivalent to a ‘minus’ subscript.

In the case where the applied field is strong enough to approach saturation magnetization (and there is a unity structure factor), the SANSPol intensities with an imperfect polarization efficiency can be reduced to 

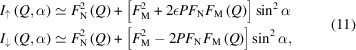

where *P* is the beam polarization, α is the angle between the field and the scattering vector, and *F*_N_ *F*_M_ is the nuclear–magnetic interference term (or ‘cross term’), which can be determined explicitly from the difference between *I*_↑_ and *I*_↓_ (Michels, 2014 ▸). We assume that the spin-flipper efficiency ε ≃ 1.

### 2.2. Detector simulation

Typically, one of two approaches are followed when it comes to treating the finite resolution of SANS instruments, either to smear the simulated intensity or to desmear the experimental intensity. Desmearing typically involves an iterative process to find a model intensity that corresponds to the experimental intensity after processing with the instrument resolution function, but this can be computationally intensive and prone to introducing artefacts into the data due to the sensitivity to initial parameter inputs and choice of algorithm (Vad & Sager, 2011 ▸). Therefore, we take the approach of smearing the simulated intensity by the instrument resolution function.

The instrument resolution is calculated in different ways, depending on the instrument. For a monochromatic pin-hole SANS instrument [*e.g.* KWS-1/2 at the Jülich Centre for Neutron Science (JCNS) at Heinz Maier-Leibnitz Zentrum (MLZ)], close to the beamstop, the resolution is mostly determined by geometric considerations (*i.e.* detector and collimation distances, sample and collimation aperture sizes, and detector pixel size); but away from the beamstop, wavelength spread dominates (Pedersen *et al.*, 1990 ▸; Barker & Pedersen, 1995 ▸). On a two-dimensional detector, the resolution has two components: one parallel to the **Q** vector (σ_*∥*_) and one perpendicular (σ_⊥_). The perpendicular resolution encodes the geometric factors, while the parallel component depends on both the geometry and the wavelength spread.

Smearing is performed by processing the simulated cross section by the instrument resolution function (Pedersen *et al.*, 1990 ▸; Mildner *et al.*, 2011 ▸) as follows: 

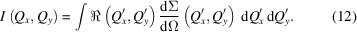

Here, the resolution function is a two-dimensional Gaussian function: 

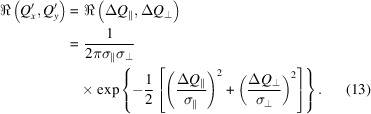

Δ*Q*_*∥*_ and Δ*Q*_⊥_ (the parallel and perpendicular components of the change in scattering vector, respectively) are derived from **Q** = (*Q*_*x*_, *Q*_*y*_) and 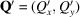 as follows: 

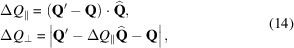

where 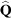 is the unit vector parallel to **Q**. Smearing acts to smooth the simulated data in a way relevant to the experimental configuration and allows the experimentally detected *Q* space to be decoupled from the simulated *Q* space. The added flexibility means that the *Q* space onto which the simulated cross section is broadcast can have both a higher resolution and a wider range than the experimental detector. Smearing is a computationally expensive step in the process, but time can be saved by pre-computing the resolution function, 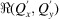 , and caching the result as *NumPy* arrays.

### 2.3. Functional overview of algorithm

**Figure 1:**
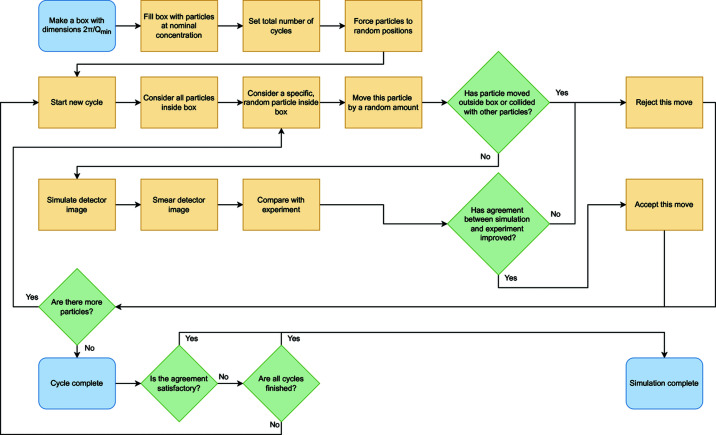
A flow diagram showing a functional overview of the algorithm process and underlying decision making.

A functional overview of how the algorithm works is shown in Fig. 1 ▸. The first step is to construct one or more physical boxes with dimensions set by 2π/*Q*_min_, where *Q*_min_ is the smallest *Q* value on the detector. Each box is filled with particles up to the nominal concentration (set as the known concentration of the experimental sample) and all particles are forced to random positions to simulate a well dispersed suspension, checking for the absence of collisions. If the particle concentration is too high, finding a well dispersed starting configuration within the bounds of the box becomes improbable and an error will be raised. We also find that loading times and initial collision checks become problematic when the number of particles per box exceeds ∼1500. For magnetic simulations, each particle is initially assigned a magnetization vector that points in a random direction but has a magnitude given as an input setting. The number of simulation ‘cycles’ is input at the start of the simulation.

At each iteration of the simulation, an action, chosen at random, is performed on a single random particle. Following Musino *et al.* (2018 ▸), actions can entail a small perturbation to a particle’s position or a jump to be adjacent to another particle. Other actions are also possible, including an orbit around a neighbouring particle, maintaining a constant separation, or a jump to a random empty position, or a physical rotation of the particle or a coherent rotation of the particle’s magnetization vector. An action may also involve varying the scale of the simulated intensity. A ‘cycle’ is considered complete when all particles have been acted upon once, at which point a new cycle is started.

After each action, the simulation decides if the action is accepted or rejected. First, the simulation checks that the action is physically accepted (*i.e.* all parameters are still within bounds and no particles impinge on each other). The simulated detector image is generated, smeared and compared with the experimental intensity. The model fitness can be determined in a number of ways but conventionally a reduced chi-squared is used, where 

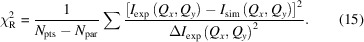

Here, *I*_exp_(*Q*_*x*_, *Q*_*y*_) is the experimental intensity, Δ*I*_exp_(*Q*_*x*_, *Q*_*y*_) is the experimental uncertainty, *I*_sim_(*Q*_*x*_, *Q*_*y*_) is the simulated intensity after smearing, *N*_pts_ is the number of points on the detector and *N*_par_ is the number of free parameters, which is typically neglected since, for a two-dimensional detector, we assume that *N*_pts_ ≫ *N*_par_. The simulated intensity may also be calculated by averaging the intensity (after smearing) from multiple independent boxes, each containing the same number of particles.

The goal of the simulation is to minimize 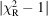 . The simulation is compatible with any number of minimizing algorithms, but a modified Metropolis–Hastings algorithm is typically used (*i.e* a simulated anneal). If a simulation step results in a negative change to the reduced chi-squared, 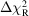 , the step is instantly accepted. The step can also be accepted if a random number generator produces a value between 0 and 1 that is less than 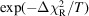 , where *T* is the temperature of the simulated anneal. The annealing schedule for *T* is set depending on the cycle number and type of simulated anneal. For example, a fast anneal goes as *T* = *T*_0_/*k* (where *T*_0_ is the starting temperature and *k* is the cycle number) (Szu & Hartley, 1987 ▸), while a very fast simulated anneal will set 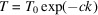 , where *c* is a rate-controlling constant (Ingber, 1989 ▸). Setting *T* = 0 simplifies to a ‘greedy’ algorithm, where only negative values for 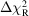 lead to acceptance. Once all cycles are completed or a satisfactory fit is obtained (*i.e.* 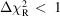 ), the simulation is terminated. If a user is unsatisfied by the result of a simulation (for example, they may wish to perform more cycles), a new simulation can be loaded from the state of a previously completed one.

The code base is written in Python 3.8 and underpinned by open-source data science libraries available on the Python Package Index (including *NumPy* and *Pandas*; The pandas development team, 2022 ▸). A typical simulation consisting of 100 particles and 200 cycles can be completed on a computer with an Intel Core i9 processor and 16 GB RAM in 152 min.

### 2.4. Structural overview of algorithm

**Figure 2:**
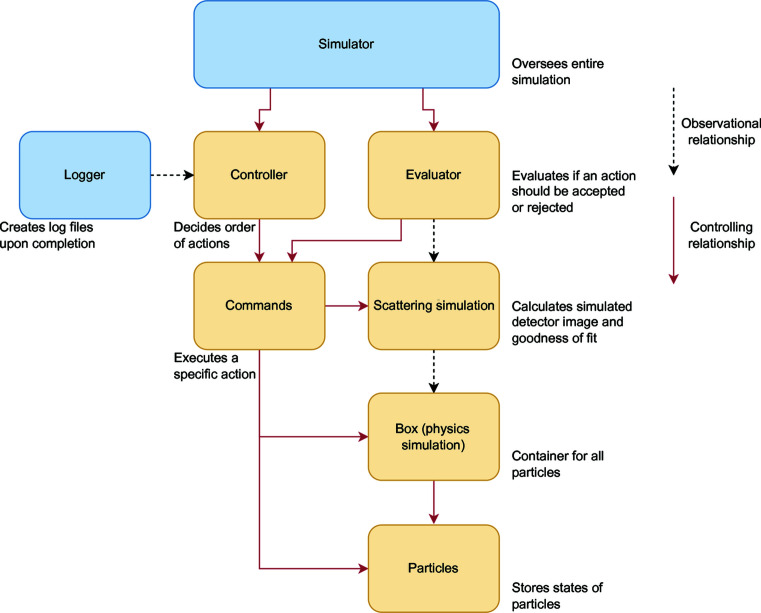
A relational chart showing how different structural components in the algorithm interact.

Structuring the code base to be mostly object orientated allows the algorithm to be assembled from modular flexible components that can be readily modified and substituted. Components and classes have been designed to have a single responsibility where feasible and as few dependencies as possible. In this section, we briefly describe the responsibility of each class or component (Fig. 2 ▸).

Individual particles are represented by an instance of a particle object, which stores information about the state of the particle, including its position, orientation and physical parameters. A cache of form amplitudes is also stored here, in the form of *NumPy* arrays. The box object stores a list of particles, and tracks if the particles are inside the box and in a physically acceptable configuration, free from collisions. The scattering simulation is responsible for performing the calculation of detector intensities described in Sections 2.1 and 2.2, and also for determining the goodness of fit with the experimental intensity.

The state of any of these components can only be modified by a command. A command encapsulates an action, performed either on the particle or on the scattering simulation, that changes the state of the simulation. The controller is responsible for maintaining a ledger of commands and updates the state of the simulation depending on the current command. The evaluator looks at the scattering simulation before and after a command has been performed, adjudicates if the command is acceptable, and then updates the state of the command to reflect whether its action has been accepted or rejected. Finally, the simulator oversees the entire simulation by iterating through commands in the controller and coordinating the evaluator.

By structuring the code base to be modular, a high degree of flexibility is enabled. Different components can be substituted with others using slightly different implementation details, and, provided the components expose the same protocol, they can be substituted without loss of functionality.

### 2.5. Experiment methods

SANS experiments were carried out at the KWS-1 instrument operated by JCNS at MLZ in Garching, Germany (Feoktystov *et al.*, 2015 ▸; Frielinghaus *et al.*, 2015 ▸). The samples were dispersed in a mixed protonated/deuterated toluene solution inside a quartz Hellma cell with a 1 mm path length, and set inside a 3 T superconducting magnet, with the samples measured at room temperature. The incident neutron wavelength λ was 5 Å and the wavelength spread Δλ/λ was 10%. The incident neutron polarization was set using a supermirror polarizer with a radio-frequency spin flipper. The sample-to-detector distance was varied between 2 and 20 m, to cover a *Q* range of 0.002–0.5 Å^−1^. Data reduction was performed using the *QtiKWS* software provided by JCNS, correcting for detector and polarizer efficiencies along with background, cell and solvent contributions. The intensity was brought to an absolute scale using plexiglass as a secondary standard. The final output was intensity, *I*, and intensity uncertainty, Δ*I*, as a function of *Q*_*x*_ and *Q*_*y*_. The parallel and perpendicular components of the instrument resolution, σ_*∥*_ and σ_⊥_, were also calculated as part of the data-reduction process.

Laboratory SAXS measurements were performed on the GALAXI instrument, which is operated by JCNS, Forschungszentrum Jülich (Kentzinger *et al.*, 2016 ▸). The samples were dispersed in toluene and filled in borosilicate capillaries with a 2 mm path length. The wavelength was 1.34 Å and the sample-to-detector distance was 3.5 m, covering a *Q* range of 0.004–0.1 Å^−1^. Full details about the SANS and SAXS experimental results are described by Nandakumaran *et al.* (2021 ▸).

## 3. Results and discussion

**Figure 3:**
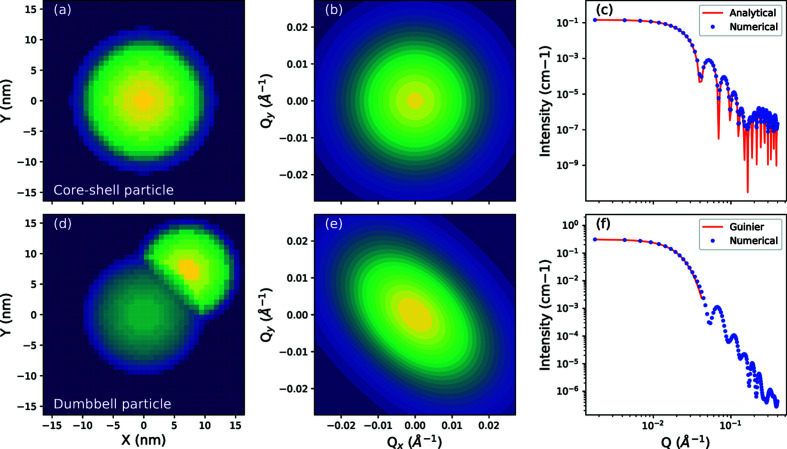
Numerical form-factor simulations. The top row shows (*a*) a real-space projection of the scattering length density of a core–shell particle, (*b*) the square of the modulus of the particle form amplitude as a function of *Q*_*x*_ and *Q*_*y*_, and (*c*) the radial average of the scattering intensity compared with the analytical profile expected from equation (3). The bottom row (*d*)–(*f*) shows the same distributions for a dumbbell particle. A fit to a Guinier approximation is shown for comparison.

Fig. 3 ▸ shows how numerical form factors can be calculated by projecting the scattering length density of a particle onto a two-dimensional real-space grid. The pixel size in Figs. 3 ▸(*a*) and 3 ▸(*d*) is 0.4 × 0.4 nm. A higher pixel density results in a scattering intensity that is closer to the analytical expression, but at the expense of computation time. Given the resolution and *Q* range of a typical SAS instrument, a pixel size of approximately this magnitude is sufficient for most applications. A core–shell particle is shown in Fig. 3 ▸(*a*). The dumbbell particle shown in Fig. 3 ▸(*d*) is notable for the presence of an excluded volume in the offset particle, which is typical for dumbbell nanoparticles in which a particle is grown on a spherical seed (Wang *et al.*, 2009 ▸). The form factor for this type of advanced particle geometry is not trivial to solve analytically (Obeidat *et al.*, 2015 ▸). The form factors shown in Figs. 3 ▸(*b*) and 3 ▸(*e*), given by |*F*_*i*_(*Q*_*x*_, *Q*_*y*_)|^2^, are determined by considering the discrete Fourier transform of the SLD shown in Figs. 3 ▸(*a*) and 3 ▸(*d*), respectively. For a non-spherical particle [such as the dumbbell particle in Fig. 3 ▸(*d*)], the discrete Fourier transform in two dimensions depends on the particle orientation. The resultant radial averages of the two distributions are shown in Figs. 3 ▸(*c*) and 3 ▸(*f*), with normalization performed by scaling based on a single particle in a box with dimensions determined from the *Q*_min_ of the two-dimensional images. The numerical distribution for the core–shell particle shown in Fig. 3 ▸(*c*) is in good agreement with the analytical profile expected from equation (3). A fit of the dumbbell profile to a Guinier approximation (Jacques & Trewhella, 2010 ▸) is shown in Fig. 3 ▸(*f*), obtaining a radius of gyration of 9.85 nm. Given that the radii of the simulated dumbbell components are 9.0 and 8.0 nm, and the centre-to-centre distance is 10.0 nm, a cylinder that encloses this dumbbell would have a radius of gyration of 10.06 nm.

**Figure 4:**
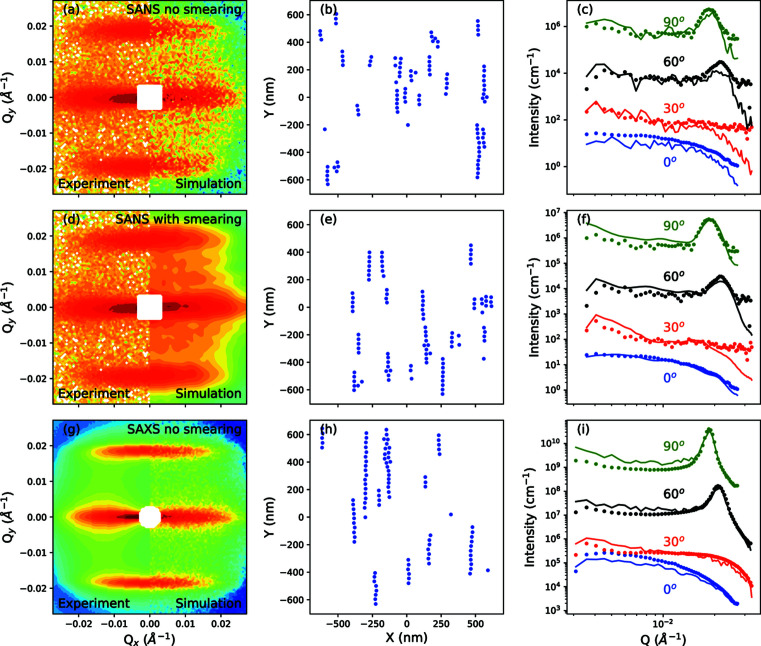
A comparison of the output of the reverse Monte Carlo algorithm with and without detector smearing applied to the simulated intensity. Comparisons are between the experimental intensity (left) and the final simulated intensity (right): (*a*) SANS with no smearing, (*d*) SANS with smearing and (*g*) SAXS with no smearing. (*b*), (*e*) and (*h*) show the particle distributions in the *xy* plane that yielded the final simulated intensities in (*a*), (*d*) and (*g*), respectively. (*c*), (*f*) and (*i*) show sector analyses of the data shown in (*a*), (*d*) and (*g*), respectively, with the symbols representing experimental data and the lines displaying the simulations. Each profile is shifted by a factor of 10^2^ compared with the one below. The field direction is parallel to the *y* axis. The experimental data set is based on results described by Nandakumaran *et al.* (2021 ▸)

Fig. 4 ▸ shows the effect of applying a smearing function to the simulated intensity at every step of the algorithm. The SANS experiment was performed on a solution of spherical iron oxide nanoparticles dispersed in toluene under an applied field of 2.2 T. The SAXS experiment was performed with the applied field set to 0.9 T (the highest available field for this instrument). The particle form-factor parameters and polydispersity were determined by previous experiments, with a nominal volumetric concentration fraction of 1.7 × 10^−3^ (Nandakumaran *et al.*, 2021 ▸). The simulation used 100 particles with a size distribution that followed the experimental polydispersity. For these simulations, particle motions were restricted to the *xy* plane. Qualitatively, the chains simulated by the SAXS data are longer, which is attributed to the higher resolution of the SAXS instrument. The final 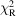 without the smearing algorithm for the SANS data set was 6.75 after 200 cycles, contrasting with 2.52 with the smearing algorithm for the same number of cycles, demonstrating that a better fit is obtained when the smearing algorithm is used. The final 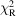 for the SAXS data set was relatively high (979), though this is attributed to a possible underestimation of the intensity uncertainty. The sector analyses in Figs. 4 ▸(*c*), 4 ▸(*f*) and 4 ▸(*i*) were taken by averaging over segments between α ± 5°, where α is the angle with respect to the *Q*_*x*_ axis in this case.

**Figure 5:**
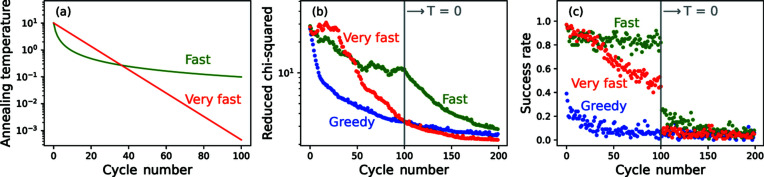
Algorithm performance as a function of different annealing schedules. (*a*) Annealing temperature for fast and very fast simulated anneals. After 100 cycles, the temperature is set to 0 for all schedules. The annealing schedule for the greedy algorithm is not shown as the temperature is always 0. (*b*) The 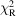 values at the end of each cycle. (*c*) The success rate for each cycle.

The performance of a set of different simulated annealing schedules is shown in Fig. 5 ▸. The target of each anneal was to fit the SANS data set shown in Fig. 4 ▸, with the smearing function turned on at every step. Three different annealing schedules were used: a fast simulated anneal starting with an annealing temperature of 10 (chosen as an arbitrarily large value), a very fast simulated anneal with the same starting temperature and a rate-controlling constant of 0.1 per cycle, and a greedy anneal with zero temperature for every cycle. The simulations were run for 200 cycles and, in every case, the annealing temperature was set to zero after 100 cycles. Fig. 5 ▸(*a*) shows the annealing schedule for the fast and very fast simulated anneals. The final output of each algorithm is displayed in Fig. 5 ▸(*b*), with the very fast anneal achieving the best fit quality after 200 cycles (by a small amount). The success rate of each cycle is shown in Fig. 5 ▸(*c*), defined as the ratio between the number of successful steps per cycle and the total steps per cycle. Here, the success rate falls rapidly for the greedy algorithm, while, for the fast schedule, it remains relatively high until the annealing temperature is turned off, suggesting that the temperature is too high to effectively distinguish moves that improve the fit. Implementing an adaptive simulate anneal, in which the temperature is adjusted according to the success rate of the most recent cycle, could be a focus for future development.

**Figure 6:**
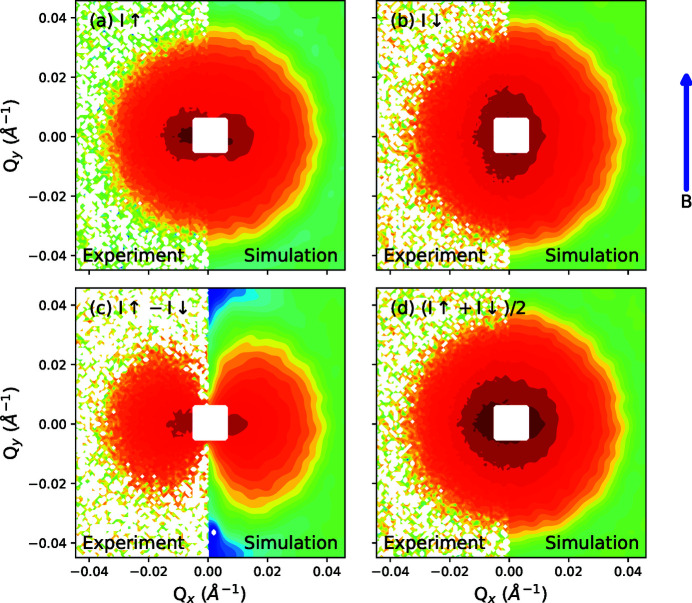
Simulated and experimental SANSPol intensities measured from 20 nm iron oxide nanoparticles at 3 T. (*a*) The spin-up detector image. (*b*) The spin-down detector image (*c*) Intensity difference between spin up and spin down. (*d*) The unpolarized intensity, recovered by averaging the two SANSPol intensities. The field direction (parallel to the *y* axis) is indicated by the blue arrow.

The effectiveness of the algorithm to model SANSPol data is shown in Fig. 6 ▸. The experimental data were collected by scattering from 20 ± 1.8 nm iron oxide nanoparticles dispersed in toluene, with the incident neutron beam polarized in one of two possible states. The simulations were performed by fitting the spin-up and spin-down detector images [Figs. 6 ▸(*a*) and 6 ▸(*b*), respectively] simultaneously, with 100 particles following a size distribution determined by experiments in a nominal volumetric concentration fraction of 0.72 × 10^−3^ and an initial (random) magnetization vector with a given magnitude. For the purposes of this simulation, an additional action involving a small coherent rotation of a given particle’s magnetization vector was included with all other actions previously described. An additional parameter to linearly scale all magnetic scattering contributions was also included (and varied) during the simulation. Multiple starting magnetization values were tested but we obtained the best agreement when we used a starting value of 279 kA m^−1^, which was estimated from experimental magnetic measurements reported previously (Nandakumaran *et al.*, 2021 ▸). In this case, the cycle number was initially set for 200 cycles but the simulation was terminated after 91 cycles, once 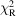 dropped below 1. A less satisfying fit (not shown here) was obtained by setting the starting magnetization value to 470 kA m^−1^, which is approximately the saturation magnetization of iron oxide (Margulies *et al.*, 1996 ▸). The intensity profiles did not show a strong influence from a structure factor.

**Figure 7:**
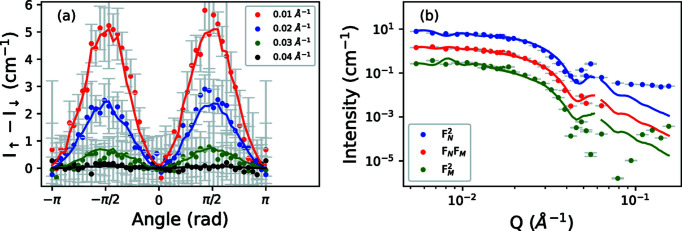
(*a*) Angle dependence of the ‘difference’ detector image shown in Fig. 6 ▸(*c*), at various *Q* values. The symbols represent experimental data, while the lines show simulated data. (*b*) The nuclear, magnetic and cross-term intensities determined by fitting the angle dependencies of the difference and unpolarized intensities to equations (11). Symbols and lines represent experiment and simulation, respectively.

Fig. 7 ▸(*a*) shows the angle dependence of the intensity of the *I*_↑_ − *I*_↓_ detector image shown in Fig. 6 ▸(*c*). If we assume that the system approaches saturation, the angle dependence of the intensity should be well described by the 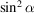 expression given in equations (11). The nuclear and magnetic scattering intensities are separated by fitting 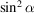 to the *I*_↑_ − *I*_↓_ detector image across the measured *Q* range and recovering the magnetic scattering from 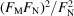 (Wiedenmann, 2000 ▸). On the basis of refinements of the nuclear and magnetic scattering intensities, we estimate a core diameter of 20.3 nm and a magnetization of 293 kA m^−1^, in good agreement with the magnetic measurements.

**Figure 8:**
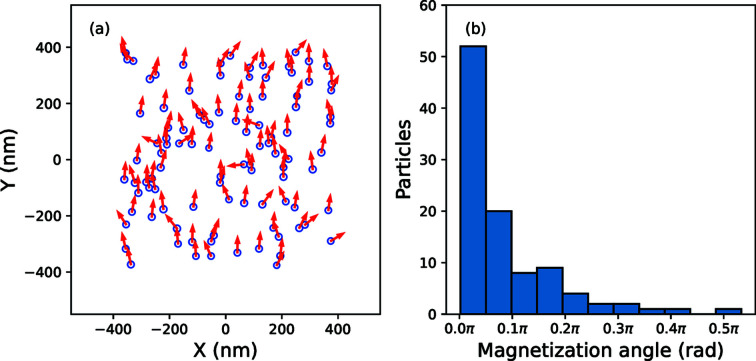
(*a*) The symbols show final particle positions that model the SANSPol intensities in Fig. 6 ▸, while the arrows indicate the final magnetization vectors. (*b*) A histogram showing the distribution of angles between the final magnetization vectors and the direction of the applied field, within the 100 particle ensemble.

The distribution of particle magnetization vectors and positions is represented in Fig. 8 ▸(*a*). The net magnetization of the final distribution can be determined from the total net moment as follows: 

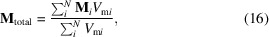

where *V*_m*i*_ is the magnetic volume of a particle *i* (typically the volume of the iron oxide core) and **M**_*i*_ is the particle magnetization vector. For this distribution, **M**_total_ has a magnitude of 268 kA m^−1^. In comparison, the net magnetization of the initial (random) configuration had a magnitude of 11 kA m^−1^, coincidentally pointing mostly antiparallel to the field direction. The orientational distribution is displayed in Fig. 8 ▸(*b*), which shows a histogram of the angles made between the particle magnetization vectors and the direction of the field. This shows that the majority of particles are aligned to within π/10 rad of the field direction. We found satisfactory agreement by relying on values for the saturation magnetization based on measurements with supplementary techniques, which is important given that this value for nanoparticles is often reduced when compared with bulk saturation (Goss, 1988 ▸; Kodama, 1999 ▸; Nedelkoski *et al.*, 2017 ▸). However, we recognize that there may also be cases where a good fit may only be achieved by also allowing the radius of the magnetic volume to vary during the simulation, along with the magnitude of the particle magnetization. This would be consistent with experimental findings that the magnetic core size and magnetic SLD are field dependent, and not necessarily fixed to the physical size of the oxide phase (Zákutná *et al.*, 2020 ▸).

## 4. Conclusions

We have reported on a reverse Monte Carlo algorithm for simulating the two-dimensional detector image observed during either a small-angle neutron scattering or a small-angle X-ray scattering experiment. The algorithm works by considering an ensemble of particles in a box and moving the particles in an iterative manner until the simulated scattering intensity matches an experimentally acquired image. Particle polydispersity and the finite resolution of small-angle scattering instruments are intrinsically accounted for within the simulations. Scattering simulations are underpinned by cross sections based on the Moon, Riste and Koehler equations, allowing simulations to take advantage of the sensitivity of small-angle neutron scattering experiments to neutron polarization and magnetic scattering, which is one of the greatest benefits of the experimental technique. The ability to account for two-dimensional scattering intensities and the neutron polarization state to model the magnetic scattering contribution distinguishes the present algorithm from previous numerical models, which have typically focused on one-dimensional scattering profiles from non-magnetic entities. Smearing of the detector image using a process underpinned by the experimental configuration is also featured, and accelerated by caching pre-calculated resolution functions.

The algorithm can be used to compute fully numerical form factors, but is also compatible with analytical expressions for particle form factors, by specifying functions that calculate the form amplitude from the scattering vector. However, the most effective use of the algorithm is to model structure factors, particularly those observed in experiments where particles self-assemble into structures that are either anisotropic or short-range ordered. Because the code base is arranged in an object-orientated structure in a modern open-source programming language, Python 3, the algorithm is highly flexible and could be readily adapted for a range of possible applications.

A maintained repository containing the full code base is available at https://github.com/lestercbarnsley/SasRMC and is released for free under the MIT license. We encourage the community’s participation in future development.
